# Benefits of rice seed priming are offset permanently by prolonged storage and the storage conditions

**DOI:** 10.1038/srep08101

**Published:** 2015-01-29

**Authors:** Saddam Hussain, Manman Zheng, Fahad Khan, Abdul Khaliq, Shah Fahad, Shaobing Peng, Jianliang Huang, Kehui Cui, Lixiao Nie

**Affiliations:** 1 National Key Laboratory of Crop Genetic Improvement, MOA Key Laboratory of Crop Ecophysiology and Farming System in the Middle Reaches of the Yangtze River, College of Plant Science and Technology, Huazhong Agricultural University, Wuhan, Hubei 430070, China; 2 Department of Agronomy, University of Agriculture, Faisalabad-38040, Punjab, Pakistan

## Abstract

Seed priming is a commercially successful practice, but reduced longevity of primed seeds during storage may limit its application. We established a series of experiments on rice to test: (1) whether prolonged storage of primed and non-primed rice seeds for 210 days at 25°C or −4°C would alter their viability, (2) how long primed rice seed would potentially remain viable at 25°C storage, and (3) whether or not post-storage treatments (re-priming or heating) would reinstate the viability of stored primed seeds. Two different rice cultivars and three priming agents were used in all experiments. Prolonged storage of primed seeds at 25°C significantly reduced the germination (>90%) and growth attributes (>80%) of rice compared with un-stored primed seeds. However, such negative effects were not observed in primed seeds stored at −4°C. Beneficial effects of seed priming were maintained only for 15 days of storage at 25°C, beyond which the performance of primed seeds was worse even than non-primed seeds. The deteriorative effects of 25°C storage were related with hampered starch metabolism in primed rice seeds. None of the post-storage treatments could reinstate the lost viability of primed seeds suggesting that seeds become unviable by prolonged post-priming storage at 25°C.

Rice (*Oryza sativa* L.) is the prominent cereal foodstuff and staple food for more than half of the world's population, especially in tropical Latin America and East, South and Southeast Asia. It is grown under wide range of environments covering approximately 11% of world arable lands^1^. Direct seeding of rice in aerobic cultures has emerged as resource conservation technology that saves water, labor, and fuel spent on puddling in transplanted rice cultures^2^. However, poor germination has been one of the obstacles in its adoption under field conditions^3^. Various seed priming techniques including hydropriming, osmopriming, hormonal priming, nutrient priming and chemical priming are employed in rice^4^. In rice crop, priming provides a vigorous ‘head start’ that typically exhibit faster and uniform emergence, accomplish better stand establishment, and gives high yields^4^. These attributes have practical agronomic implications, notably under stressful environments during germination^5^. Nonetheless, the rice growers often find it difficult to treat rice seeds before sowing. Therefore, there is a growing interest in the seed industry to market treated seeds that might achieve good crop stands not only under normal conditions but also enhance the tolerance of plants under adverse field conditions. However, prolonged storage of treated seeds may be critical in marketing of primed rice and other crops' seeds.

Seed priming reduces the longevity of primed seeds enormously as compared with non-primed seeds^6^,^7^,^8^,^9^. Primed sweet corn seed exhibited poor germination and seedling growth performance after 3 months of storage at 25°C than non-primed seed^7^. Delayed and lower germination was recorded in primed tomato seeds, when stored at 30°C for 6 months as compared with the control^6^. Primed lettuce seeds were particularly prone to reduced longevity relative to non-primed seeds when stored under high temperature and high moisture conditions^8^,^9^. Even under mild storage conditions (45°C at 50% relative humidity), primed lettuce seeds exhibited slower and less synchronous germination than the non-primed seeds in as little as 14 d^10^. Nevertheless, it has been reported that seed priming prior to storage improved the longevity of seeds^11^,^12^. Dearman *et al.* reported the priming before storage delayed the loss of viability in onion, but post-storage priming had no effect on viability^13^. The primed and dried onion seeds maintained improvements in germination even after 18 months storage at 10°C^13^. Chiu *et al.* observed that primed sweet corn seeds stored at 10 or −80°C recorded higher longevity, and showed better germination and vigor responses than non-primed seeds even after 12 months storage^7^. Rajjou and Debeaujon concluded that such variations in longevity are governed by environmental and genetic factors such as storage temperature, seed moisture content, and seed quality^14^. High seed moisture and high temperature are known to further accelerate the rate of seed deterioration^15^.

Reduced longevity of primed seeds can be partially restored by post-storage treatments such as, heat shock and dehydration treatment^16^ and post-storage humidification or priming^17^. Butler *et al.* found that repeated priming after storage assuaged the damaging effects of storage on seed viability^12^. Even in the natural environment, macromolecules within the seed tissues incur some damage through normal metabolism; and repair is possible, if the damage accumulated is not too severe. Most of the studies pertaining to investigate the longevity of primed seeds during storage have been conducted under artificial ageing^8^,^9^,^18^, where high temperature and humidity conditions are normally used. Recently, Schwember and Bradford reported that longevity of seeds stored in conventional and controlled deterioration (CD) conditions was rarely correlated for both non-primed and primed seeds^19^. They suggested that CD tests at elevated temperature and moisture may not be predictive of commercial seed storage conditions, and may lead to over estimation of results^19^. It is imperative to test longevity under more moderate storage conditions and developing methods to predict potential storage life well before the loss of viability is even more important^20^.

However, reports about the storage potential of primed rice seeds are limited. A real understanding of such changes in pre-treated rice under varying storage environments are of great ecological, agronomic and economic significance. The aims of the study were (1) to investigate the effects of storage temperature (25°C and −4°C) on germination and seedling growth performance of primed and non-primed rice seeds, (2) to determine possible loss of viability of primed rice seeds during storage, and (3) to explore the effects of post-storage treatments (re-priming and heating) on viability of primed rice seeds.

## Results

### Experiment 1: Germination and growth response of primed and non-primed rice seeds after 210 days of storage at 25°C or −4°C

#### Seed germination

**Figure 1:**
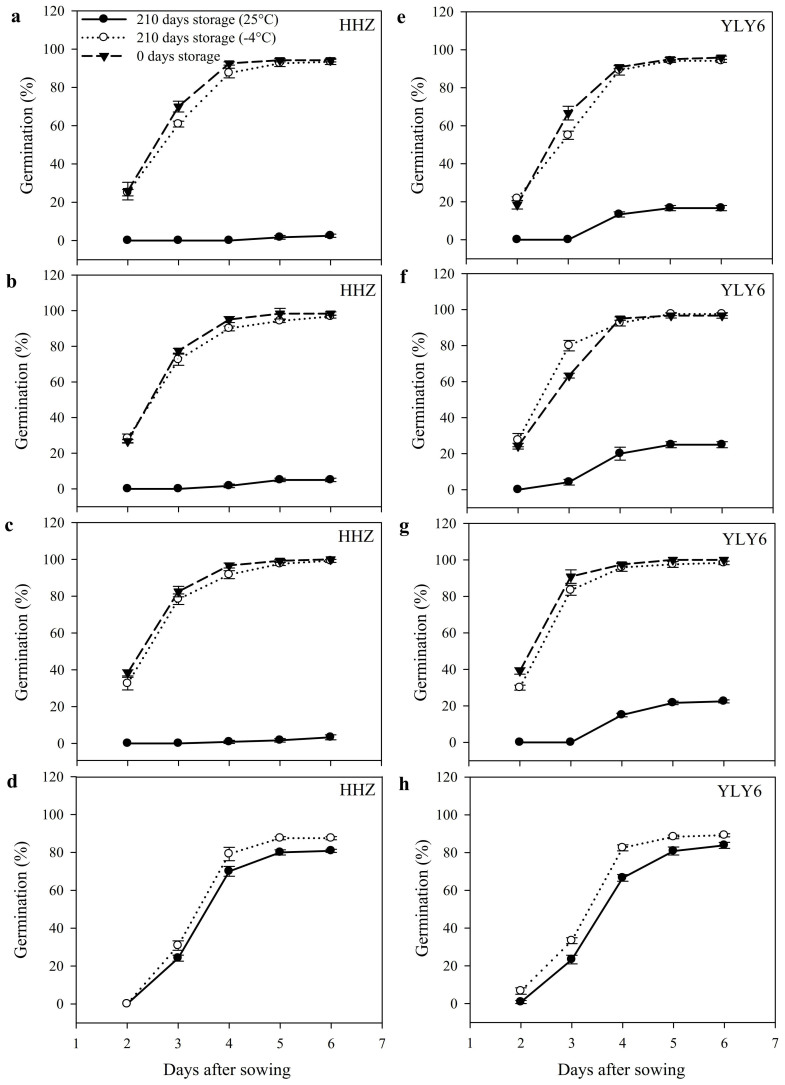
Variations in germination dynamics of primed and non-primed rice seeds after 210 days of storage at 25°C or −4°C in Experiment 1. (a, e) Hydropriming. (b, f) 10% polyethylene glycol priming. (c, g) 0.5 mmol L^−1^ spermidine priming. (d, h) non-primed seeds. (HHZ: Huanghuazhan, YLY6: Yangliangyou-6, DAP: days after priming). Fresh primed seeds were sown in 0 DAP treatment. Only two curves have been shown in non-primed seeds (d, h), because same seed was used for “0 days storage” and “210 days storage at −4°C” treatments. Since the germination was constant after 6 days of sowing (DAS) in all treatments, therefore, germination data up till 6 DAS have been presented. Error bars indicate standard error (n = 4).

Temporal data regarding germination dynamics of primed and non-primed rice seeds after 210 days of storage are presented in Figure 1. Pronounced variations were recorded between primed and non-primed seeds of both rice cultivars (Huanghuazhan; HHZ and Yangliangyou-6; YLY6) under varying storage conditions. Primed rice seeds of both cultivars were more prone to storage conditions (Fig. 1). Primed seeds stored at 25°C largely lost their viability, therefore, only 3.6% and 21.3% seeds of HHZ and YLY6, respectively, could germinate on average across different priming treatments. Nevertheless, germination of primed seeds stored at −4°C for 210 days, was almost similar with that of un-stored (0 DAP-fresh primed) seeds. Although, storage of non-primed seeds at 25°C reduced the germination of HHZ and YLY6 by 7.6% and 9.3%, respectively; nonetheless, these negative effects were much lower as compared with those observed for primed seeds (Fig. 1d & h).

Significant (*P ≤ 0.05*) effects of seed storage as well as priming treatments on germination attributes were recorded for both rice cultivars (Table 1). Seed priming significantly improved the germination percentage, germination index and vigor index as compared with non-primed seeds when recorded at 0 DAP. Spermidine- primed (Spd) seeds outperformed polyethylene glycol- (PEG) and hydro- primed (HP) seeds regarding these attributes. Germination percentage, germination index and vigor index of primed seeds stored at −4°C for 210 days, were statistically (*P ≤ 0.05*) similar with freshly primed (0 DAP seeds) in both rice cultivars. However, when primed seeds were stored at 25°C, significant (*P ≤ 0.05*) reductions in germination percentage (87.1%), germination index (94.2%) and vigor index (98.7%) were observed averaged across different priming treatments and cultivars (Table 1). Such deleterious effects of storage at 25°C were more pronounced in primed seeds of HHZ than those of YLY6. In non-primed seeds, reductions in germination percentage, germination index and vigor index after storing for 210 days at 25°C were on average 8.5%, 10.9% and 37.1%, respectively across two rice cultivars.

#### Seedling growth

**Figure 2:**
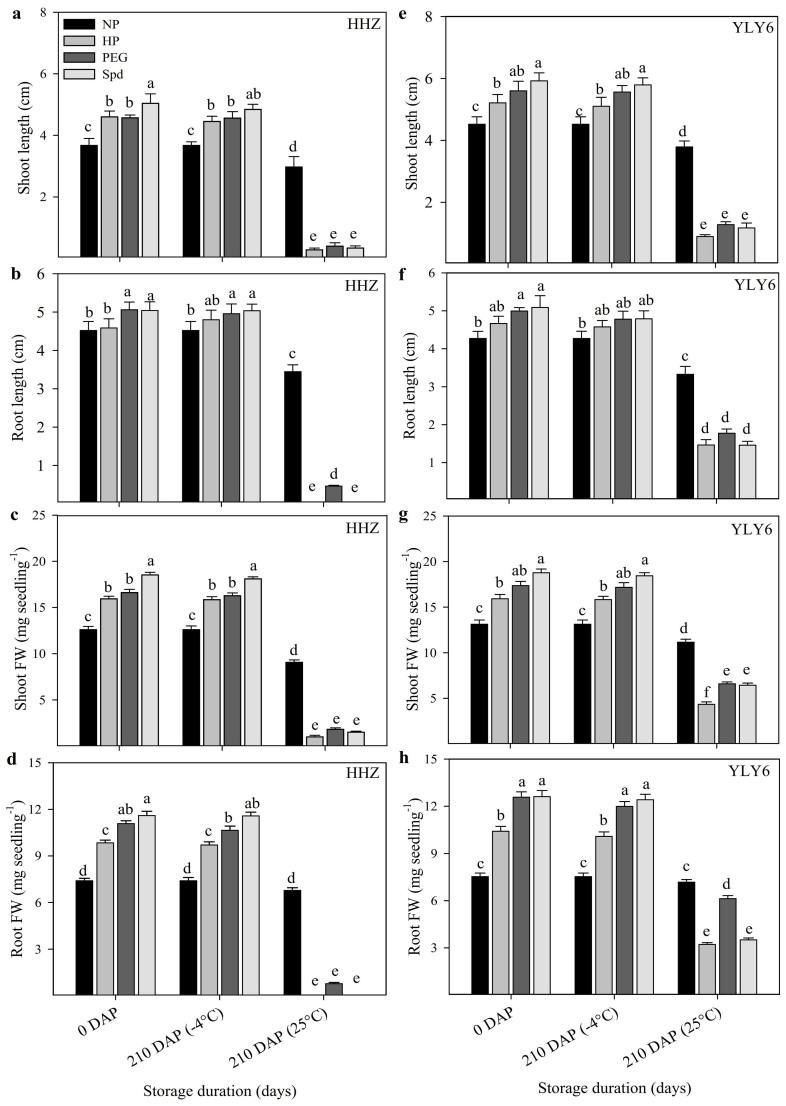
Seedling growth of primed rice seeds after 210 days of storage at 25°C and −4°C in Experiment 1. (a, e) Shoot length. (b, f) Root length. (c, g) Shoot FW. (d, h) Root FW. (NP: non-primed seeds, HP: hydropriming, PEG: 10% polyethylene glycol priming, Spd: 0.5 mmol L^−1^ spermidine priming, HHZ: Huanghuazhan, YLY6: Yangliangyou-6, DAP: days after priming, FW: fresh weight.) Fresh primed seeds were sown in 0 DAP treatment. Different lowercase letters denote statistical differences between treatments of a cultivar at the 5% level according to LSD test. Error bars above mean indicate standard error (n = 4).

Seedling growth of primed and non-primed seeds of two rice cultivars after 210 days of storage is depicted in Figure 2. Compared with non-primed seeds, significantly (*P ≤ 0.05*) higher shoot length, root length, and their fresh weights were recorded for un-stored seeds (freshly primed seed-0 DAP) and those stored at −4°C for 210 days after priming. Seedling growth of 0 DAP seeds and 210 DAP stored at −4°C was similar (Fig. 2). However, when primed seeds were stored at 25°C, significant (*P ≤ 0.05*) decrease in seedling growth of both rice cultivars was observed, and such a decrease was more pronounced in HHZ as compared with YLY6. In HHZ, the shoot length, root length, shoot fresh weight and root fresh weight of primed seeds stored at 25°C, were decreased by 92.9%, 96.9%, 91.4% and 97.4%, respectively compared with un-stored seeds averaged across different priming treatments. The respective reductions in primed seeds of YLY6 upon storage at 25°C were 81.2%, 68.1%, 66.6% and 63.9%, respectively. All the priming treatments responded in a similar manner to different seed storage treatments. In non-primed seeds, the reductions in seedling growth attributes due to storage at 25°C were <25% (Fig. 2).

#### Starch metabolism

α-Amylase activity and total soluble sugar content recorded in rice seeds (0 days after sowing; DAS) and seedling (7 DAS) varied significantly (*P ≤ 0.05*) in response to seed storage and priming treatments in both cultivars (Table 2). Significantly (*P ≤ 0.05*) higher α-amylase activity and total soluble sugars were recorded in un-stored (0 DAP) primed rice seeds and seedlings as compared with non-primed treatments. Primed seeds stored at −4°C for 210 days recorded α-amylase activity and total soluble sugar content that was similar (*P ≤ 0.05*) with 0 DAP treatments. Nevertheless, seed storage at 25°C posed a detrimental effect on both of these attributes. Averaged across different priming treatments and cultivars, storage of primed seeds at 25°C diminished α-amylase activity of rice seeds and seedlings by 48.9% and 73.8%, respectively. The total soluble sugars content in primed rice seeds and seedlings were also reduced by 59.7% and 57.3% upon storage for 210 days at 25°C. In non-primed rice seeds and seedlings, the reductions in α-amylase activity and total soluble sugar contents were <10%, when dry seeds were stored at 25°C for 210 days (Table 2).

### Experiment 2: Influence of different seed storage durations (25°C) on germination and early seedling growth of primed rice seeds

Previously in Experiment 1, we studied the germination and early seedling growth response of primed and non-primed rice seeds after 210 days of storage under different temperature regimes. Based on the results, we found that primed rice seeds stored for 210 days at 25°C largely lost their viability due to severe seed deterioration, and that was never the case when the seeds were stored at −4°C for similar time span. Furthermore, the negative effects of storage at 25°C in non-primed seeds were not as prominent as in primed seeds. Therefore, in the following Experiment 2, the durations (25°C) of seed storage were lowered to 0, 15, 30, 45 and 60 DAP to ascertain how long a primed rice seed can maintain improvements induced by priming when stored at 25°C (near ambient temperature) without losing its viability. Seed germination, early seedling growth and starch metabolism in rice seeds as well as in seedlings were investigated.

#### Seed germination

**Figure 3:**
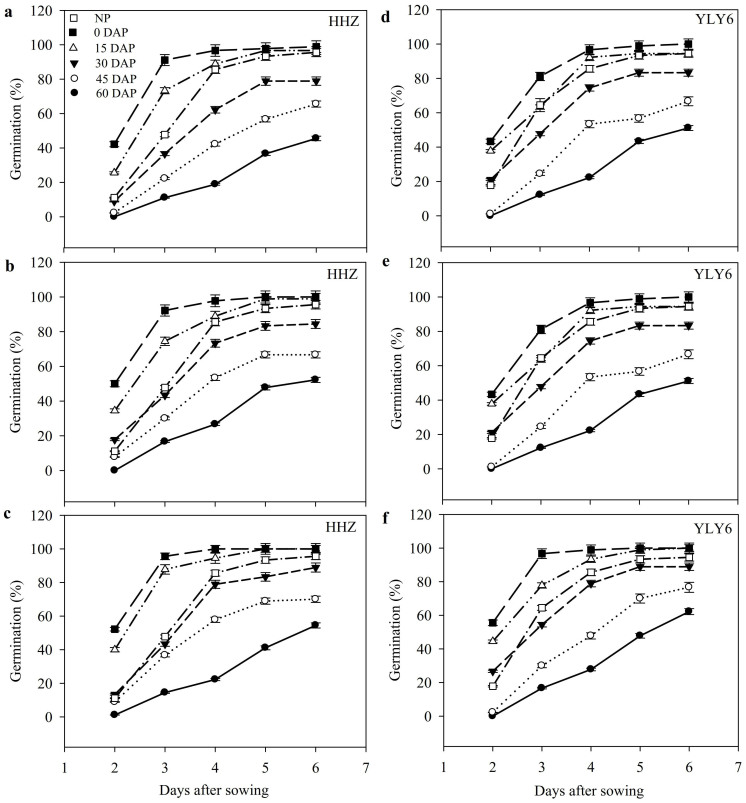
Variations in germination dynamics of primed rice seeds after 0, 15, 30, 45 and 60 days of storage at 25°C in Experiment 2. (a, d) Hydropriming. (b, e) 10% polyethylene glycol priming. (c, f) 0.5 mmol L^−1^ spermidine priming. (HHZ: Huanghuazhan, YLY6: Yangliangyou-6, DAP: days after priming. DAP: days after priming.) Fresh primed seeds were sown in 0 DAP treatment, while in NP, non-primed seeds stored at −4°C were used for comparison. Since the germination was constant after 6 days of sowing (DAS) in all treatments, therefore, germination data up till 6 DAS have been presented. Error bars indicate standard error (n = 4).

Data regarding germination dynamics of primed rice seeds after storage for 0, 15, 30, 45, and 60 days at 25°C are shown in Figure 3. All the storage durations for primed seeds starting from 15 DAP considerably decreased the rice germination when compared with 0 DAP treatments in both cultivars (Fig. 3). Increase in duration of storage reduced the germination performance of primed seeds, therefore, after 60 days of storage, only 50.7% and 57.4% primed seeds could germinate (at 6 DAS) in HHZ and YLY6, respectively as compared with near 100% germination as recorded for un-stored (0 DAP) seeds. As compared with non-primed seeds, priming had positive effect up till 15 days after storage, beyond which germination was lower than non-priming treatments in both cultivars (Fig. 3).

Evaluation of germination percentage, germination index and vigor index showed significant (*P ≤ 0.05*) variation in both rice cultivars under the influence of different storage durations of primed rice seeds and priming treatments (Table 3). In 0 DAP treatments, seed priming significantly improved the germination percentage (11.6%), germination index (33.4%) and vigor index (49.6%) as compared with non-primed seeds while the priming treatments (HP, PEG, Spd) were statistically similar (*P ≤ 0.05*) to each other. However, when primed seeds were stored at 25°C, all the germination attributes were considerably decreased particularly after 15 days of storage. When evaluated for storage duration of 60 DAP, germination percentage, germination index and vigor index were reduced by 46.4%, 71.6% and 87.6% averaged across different priming treatments and cultivars (Table 3).

#### Seedling growth

**Figure 4:**
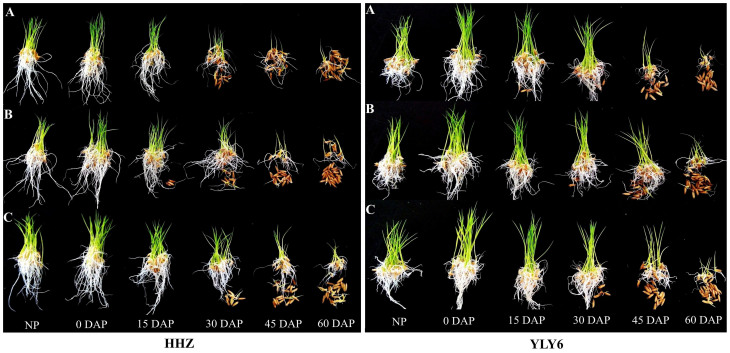
Early growth performance of primed rice seeds of two different cultivars after 0, 15, 30, 45 and 60 days of storage at 25°C in Experiment 2. (A), (B), and (C), represent the hydropriming, polyethylene glycol priming, and spermidine priming, respectively. (HHZ: Huanghuazhan, YLY6: Yangliangyou-6, DAP: days after priming.) All the pictures were taken after 7 days of sowing. Fresh primed seeds were sown in 0 DAP treatment.

**Figure 5:**
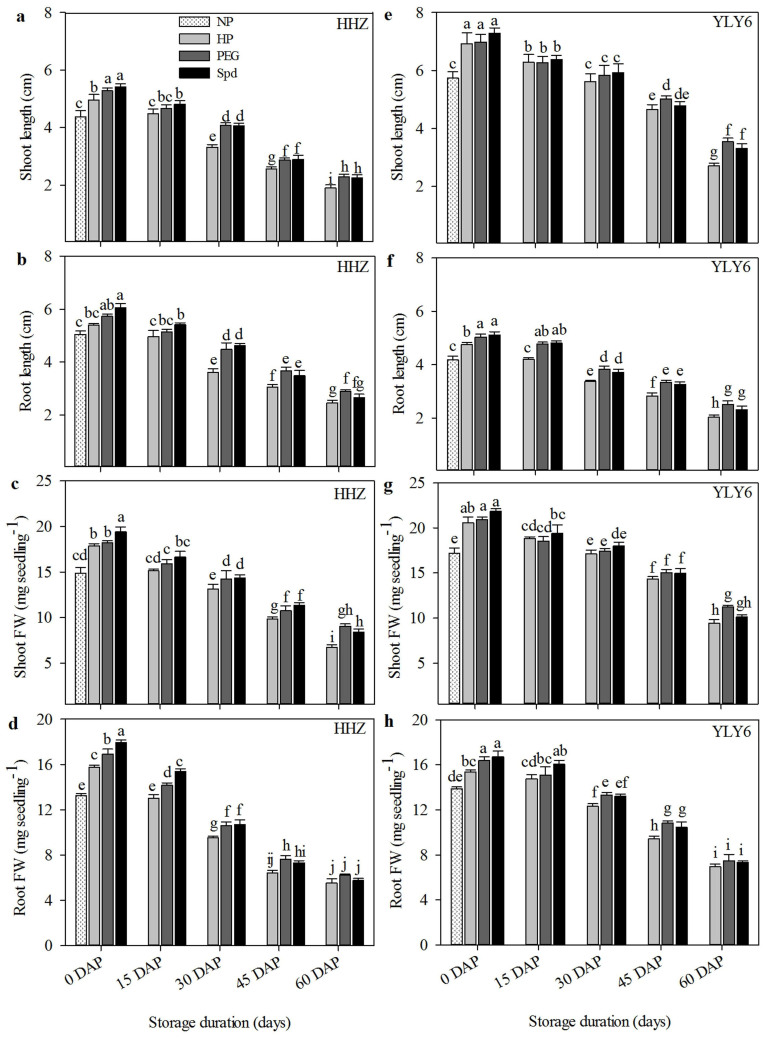
Seedling growth of primed rice seeds after 0, 15, 30, 45 and 60 days of storage at 25°C in Experiment 2. (a, e) Shoot length. (b, f) Root length. (c, g) Shoot FW. (d, h) Root FW. (NP: non-primed seeds, HP: hydropriming, PEG: 10% polyethylene glycol priming, Spd: 0.5 mmol L^−1^ spermidine priming, HHZ: Huanghuazhan, YLY6: Yangliangyou-6, DAP: days after priming, FW: fresh weight.) Fresh primed seeds were sown in 0 DAP treatment. Different lowercase letters denote statistical differences between treatments of a cultivar at the 5% level according to LSD test. Error bars above mean indicate standard error (n = 4).

A pictorial view of primed rice seed performance in response to different seed storage duration in two different cultivars is presented in Figure 4. Significant (*P ≤ 0.05*) effects of seed storage durations and priming treatments on rice seedling growth were observed in both cultivars (Fig. 5). As consistent with Experiment 1, shoot length, root length and their fresh weights decreased in both rice cultivars, when primed seeds were stored at 25°C for varying durations. Increase in duration of storage of primed seeds brought a concomitant decrease in rice seedling growth. Shoot length of HHZ cultivar decreased by 10.9%, 26.8%, 46.7%, and 58.8%, when evaluated for seeds stored for 15, 30, 45 and 60 days, respectively as compared with un-stored seed. Similar was the case for YLY6, in which 10.6%, 17.9%, 31.7% and 54.8% reductions in shoot length were observed after 15, 30, 45 and 60 days of storage, respectively. Averaged across different priming treatments and cultivars; root length, root fresh weight and shoot fresh weight were decreased by 11.0–53.5%, 10.6–60.1% and 12.1–53.7%, respectively, when seed storage duration was prolonged from 15 DAP to 60 DAP. All the seedling growth attributes recorded in 0 DAP and 15 DAP treatments were higher than non-primed seeds, when considering the average of all priming treatments and cultivars (Fig. 5).

#### Starch metabolism

Significant variation (*P ≤ 0.05*) in starch metabolism of primed rice seeds after 0, 15, 30, 45, 60 days of storage at 25°C were observed in both rice cultivars (Table 4). In un-stored seeds (0 DAP), all the priming treatments exhibited significantly (*P ≤ 0.05*) higher α-amylase activity and total soluble sugars in rice seeds and seedlings as compared with non-priming treatment. However, storage of primed seeds at 25°C even for only 15 days severely declined the α-amylase activity and total soluble sugars in rice seeds as well as seedlings emerging from primed seeds stored for such duration. When averaged across priming treatments and cultivars, α-amylase activity in rice seedlings (7 DAS) declined by 13.9%, 27.4%, 43.2%, and 63.0% as observed for storage durations of 15, 30, 45, and 60 DAP, respectively as compared with un-stored primed seeds. The α-amylase activity in rice seeds (0 DAS) was reduced in the range of 6.8–40.7% as seed storage duration was prolonged from 15 to 60 DAP. The total soluble sugars contents in primed rice seeds and seedlings depicted trends that were similar to α-amylase activity. Averaged across priming treatments and cultivars, 7.3%, 24.0%, 42.8%, and 51.8% reductions in total soluble sugars of primed rice seedlings were observed after 15, 30, 45 and 60 days of storage at 25°C, respectively. Likewise, total soluble sugars of primed rice seeds were also reduced by 2.9%, 14.4%, 28.8%, and 36.0% for aforementioned durations of storage, respectively.

### Experiment 3: Influence of post-storage treatments (re-priming and heating) on germination of primed rice seeds stored at 25°C for 210 days

In Experiment 1, seed priming followed by storage at 25°C for 210 days severely diminished the viability of rice seeds. It was postulated that post-storage treatments may reinstate the viability of primed seeds. Therefore, in Experiment 3, stored primed seeds were either re-treated with same priming agents or heated at 45°C for 24 h. Results revealed that none of the post-storage seed treatment could significantly (*P ≤ 0.05*) alter the germination of stored primed seeds (Table 5). Maximum germination (>80% in both cultivars) was recorded in non-primed seeds, which was significantly (*P ≤ 0.05*) higher than the stored primed seeds. In stored primed seeds of HHZ, not a single treatment could excel 5% germination even after re-priming or heating. In case of YLY6, germination percentage of stored primed seeds was in the range of 18–23%, and all the primed seed treatments were statistically (*P ≤ 0.05*) similar with each other (Table 5). The data of germination were also linked with the α-amylase activity and total soluble sugars in rice seedlings. After 7 DAS, there were no significant (*P ≤ 0.05*) differences among re-priming, heating and control (no post-storage treatment) treatments regarding α-amylase activity and total soluble sugar contents in rice seedlings of both cultivars. However, both of these attributes were significantly (*P ≤ 0.05*) higher in non-priming treatment, in which non-primed dry seeds were stored at 25°C for 210 days.

## Discussion

In the era of climate change, global food security is confronted by escalating food demand and endangered by dwindling water resources. This scenario has compelled the rice growers to shift to direct seeding as an alternative to current practice of transplanting in flooded soils^2^. Nevertheless, erratic germination and poor stand establishment in direct-seeded rice is a major deterrent for achieving an optimal crop growth and better productivity^3^. Seed priming has emerged as an effective and pragmatic approach for increasing seed vigor and synchronization of germination, as well as the seedling growth of rice^4^. However, storage of primed seeds is a major hurdle in success of seed priming. Although, little is known about storage potential of primed rice seeds, nonetheless, plenty of studies have previously reported the reduced longevity of primed seeds due to prolonged storage in many vegetables and field crops^6^,^7^,^8^,^9^,^10^.

In our study, storage of primed seeds at 25°C posed highly deleterious effects on germination and early seedling growth of rice. Germination and seedling growth of both rice cultivars were strongly linked with starch metabolism both in rice seeds and its seedlings. Seed storage for 210 days at 25°C severely reduced the α-amylase activity and total soluble sugars (>50%) in both rice cultivars (Table 2), possibly because starch degradation was hampered and seed reserves were highly deteriorated during storage at this temperature. The ability of plants to degrade starch into soluble sugars probably plays a key role in their ability to survive and grow faster under wide range of environments. In rice, amylase activity is highly induced during germination^21^. In Experiment 1, α-amylase activity in freshly primed seeds (un-stored) was increased by 108.7% from 0 DAS (seeds) to 7 DAS, whereas, in stored seeds (210 days at 25°C), it was increased by only 6.9%, which clearly justifies very poor germination and growth performance of stored seeds (25°C). High α-amylase activity in freshly primed seeds is also reflected through higher soluble sugar concentrations (Tables 2 and 4) and faster rate of starch breakdown in germinating primed seeds, which presumably provided the substrates necessary for generating the energy required for growth and maintenance processes. It has also been proposed that the degradation and conversion of seed reserves during germination process may be related to the increase in soluble sugar contents^22^, and therefore, might be the possible cause of higher soluble sugar contents in freshly primed seeds. Moreover, minimal increase in soluble sugar contents of stored (210 days at 25°C) primed seeds, depicts that germination process could not be initiated in seeds after such storage.

It is important to know that the primed rice seeds stored for 210 days at different storage conditions (25°C and −4°C) showed variable response in relation to germination and early seedling growth. The performance of primed seeds stored at −4°C even after 210 days, was similar to un-stored primed (0 DAP) seeds. Previously, several authors have concluded that negative effects of seed storage on primed seeds, are temperature dependent^6^,^7^,^8^,^9^. While studying sweet corn, Chiu *et al.* observed reduced seed longevity, when primed seeds were stored at 25°C for 12 months. However, storage at 10°C or −80°C improved the performance of primed seeds compared with non-primed seeds even after 12 months^7^. Buitink *et al.* stated that higher longevity of seeds stored at low temperatures might be due to extremely high viscosity and low molecular mobility of the seed cytoplasm that could prevent or inhibit many deleterious processes^23^. Whereas, with increasing temperature, the low viscosity and enhanced molecular mobility would permit certain deteriorative reactions to proceed rapidly, which are otherwise retarded in the cold storage conditions^23^.

Compared with primed seeds, seed storage at 25°C for 210 days had a little effect on longevity of non-primed seed so that performance of non-primed seeds stored at 25°C or −4°C was almost similar. These results are in agreement with Argerich *et al.*, who reported that both viability and germination of non-primed tomato seeds were unaffected by 4°C or 30°C storage for one year, or by 4°C storage in primed seeds^6^. At 30°C, however, viability and germination rate of primed seeds were reduced markedly even after 6 months of storage.

In Experiment 2, different seed storage durations starting from 0 to 60 days were tested in order to investigate that how long a primed rice seed can maintain the positive effects of seed priming, when stored near ambient temperature (25°C). Increase in storage duration depressed the longevity of primed seeds. In addition to delay and reduced germination, storage of primed seeds also resulted in meager and stunted growth of seedling. These results are in agreement with McDonald, who stated that the incidence and severity of such negative effects was increased with storage time^5^. In our study, beneficial effects of seed priming were maintained only up to 15 days of post-priming storage and the performance of primed rice seeds was even worse than non-primed seeds beyond that duration of storage. These results foretell that rice seed priming for more than 15 days prior to sowing is useless or even it may cause economic loss, if seed is stored at 25°C.

Primed and non-primed seeds stored at 25°C for 210 days had almost similar moisture contents (avg. moisture content: 9.10% and 9.05%, respectively) and received same storage conditions. However, their viability varied after 210 days of storage, e.g. total soluble sugar contents in non-primed stored seeds were reduced by only 6.20% after 210 days at 25°C storage, whilst, reductions in total soluble sugar contents of primed stored seeds after 210 days of storage at 25°C, were 59.72% on average compared with 0 DAP treatments (Table 2). Furthermore, viability of primed seeds stored at −4°C did not change even after 210 days, which suggests that seed priming along with storage at high temperature (25°C) was mainly responsible for reduced seed longevity in present study. Previously, Garcia *et al.* stated that soluble sugar contents in dry seeds (moisture contents: 7–8%) of *Caesalpinia echinata* were severely decreased after 12 and 18 months of storage at 25°C^24^. However, such negative effects were not observed, when seeds were stored under cold conditions (6°C). While working on maize, Bernal-Lugo and Leopold also reported more than 60% decrease in sugar contents of corn embryo after 90 days of storage at 30°C^25^.

Another possible factor for seed deterioration in present study might be the rise of oxygen concentration during storage that has triggered the metabolic processes in seeds at high temperature (25°C), because seeds were stored in plastic (polyethylene) bags which have high permeability to oxygen. Nonetheless, no such effects have been observed under −4°C storage, because at low temperature storage, presence of oxygen has not been shown to influence seed viability, instead at high temperature, rapid seed deterioration in viability has been documented^26^. Increase in oxygen pressure has been reported to regulate a number of physiological changes e.g. loss of food reserves (proteins and sugar contents) caused by respiration, which are associated with seed deterioration^27^. Ellis and Roberts stated that low temperature and seed moisture contents may decrease the level of respiration, thereby reducing seed viability losses, however, quantitative predictions regarding effect of oxygen levels on seed longevity are lacking^26^. Our results suggested the need of further studies to explore the influence of oxygen on longevity of primed rice seeds under different seed moisture contents and temperature regimes during storage.

Almost similar patterns of response by seeds primed with different agents in present studies to varying seed storage conditions and durations suggests the possibility for applicability of these findings to wide range of priming agents in rice crop. The growers who prime/pre-treat their own seeds several months before planting, need to know the extent of seed storage temperature and critical duration that will retain the maximum benefits of such invigoration. In the developing countries lacking appropriate seed storage infrastructure for −4°C, farmers can prime the rice seeds within 15 days prior to sowing, and can store at near 25°C temperature under dry conditions. While, the other option (to store the dry primed seeds at −4°C) can be acceptable for the farmers of developed countries and seed companies as well.

Cultivar variations were also apparent regarding their response to seed storage. After 210 days of storage at 25°C, hybrid cv. YLY6 recorded 21.3% germination; while in HHZ, the average germination was 3.6%. This suggests that along with the variations in plant species^28^, seed storage potential may also differ within a species. Further efforts can be done to screen/breed cultivars with high storage potential.

The results of Experiment 3 are contradictory to some previous reports, which reveal that viability of primed stored seeds can be partially restored by post-storage treatments^12^,^16^,^17^. Butler *et al.* found that repeated priming after storage assuaged the damaging effects on viability^12^. In present study, heating (45°C for 24 h) as well as re-priming of 210 days old primed (stored at 25°C) seeds could not reinstate the viability of aged primed seeds. This suggests that primed seeds become unviable after prolonged storage at 25°C.

In crux, seed priming is a pragmatic technique which can efficiently enhance the germination and growth of rice, nevertheless, storage of primed seeds is a major hurdle in marketing and technology transfer of this technique. In series of studies, we concluded that storage of primed seeds at 25°C beyond 15 days is deteriorative for germination and growth of rice, and such damaging effects were related with hampered starch metabolism in primed rice seeds. However, if primed rice seeds are stored at −4°C, there will be no negative effect on seed viability even after prolonged storage. Post-storage treatments could not reinstate the lost vigor of primed seeds, suggesting permanent loss of viability by post-priming storage at 25°C for prolonged period of time.

## Methods

### Plant material

Seeds of two widely grown indica rice (*Oryza sativa* L.) cultivars viz., Huanghuazhan (HHZ, inbred) and Yangliangyou6 (YLY6, hybrid) having initial germination of >95% were used in these studies. The initial seed moisture content (MC) was 9.60% (on dry weight basis). Healthy seeds selected from same seed lot were used for all the experiments. Prior to experimentation, seeds were stored in refrigerator (−4°C).

### Treatments

Seed priming treatments used in all the experiments were: hydropriming (HP), polyethylene glycol- (PEG) priming and spermidine (Spd) priming. Effective levels of PEG (10%) and Spd (0.5 mmol L^−1^) for priming solutions, were pre-optimized based on rice emergence and early seedling growth performance. Autoclaved distilled water was used for hydropriming. Seeds were primed in dark at 25°C for 24 h, with constant gentle agitation. The ratio of seed weight to solution volume (w/v) was 1:5. The priming solution was changed after every 12 h. Untreated seeds (non-primed) taken from same seed lot were used as control. After 24 h, the primed seeds were washed with distilled water for 2 mins, surface dried by blotting paper and transferred to air dry oven at 25°C for 48 h to reduce the moisture content. Dried primed (avg. moisture content: 9.10% on dry weight basis) and non-primed seeds (avg. moisture content: 9.05% on dry weight basis) were then subjected to different seed storage treatment.

In Experiment 1, primed and non-primed seeds of both rice cultivars were stored at 25°C or −4°C for 210 days. For 25°C storage treatments, seeds in plastic bags were placed in air-conditioned laboratory room with light/dark period of 12/12 and relative humidity 50 ± 5%. While for −4°C storage; similar amount of seed was placed in refrigerator. Fresh priming treatment (un-stored seed) was used for comparison. Because the differences in performance of non-primed seeds stored at 25°C or −4°C were minimal as compared to primed seeds, therefore in Experiment 2, only primed seeds were stored for 0, 15, 30, 45 and 60 days after priming (DAP), to ascertain the storage potential of primed rice seeds at 25°C. One non-primed seed treatment (seed stored at −4°C) was run for comparison. The seeds of Experiment 1 stored at 25°C were transferred to refrigerator (−4°C) for Experiment 3 at 210 DAP. In Experiment 3, old primed seeds (210 days of storage at 25°C), were subjected to post-storage treatments viz., heating and re-priming, in order to evaluate their effects on viability of primed seeds. For heating, old primed seeds were placed in oven at 45°C for 24 h to give heat-shock; while, for re-priming, previously primed seeds were retreated with same priming agent according to protocol mentioned above.

### Experimentation

All the experiments were conducted in Crop Physiology and Production Center at Huazhong Agricultural University, Wuhan, China during 2014 with same experimental procedure. Thirty healthy seeds from each treatment were evenly germinated on two layers of filter paper in 14.5 cm diameter Petri dishes. After adding 20 ml water to each replicate, Petri dishes were covered with lid and placed on steel racks in growth chamber with 12 h light period, and 30°C day: 25°C night temperature. The humidity during the course of study was maintained at 60%. Equal volume of distilled water was applied to all Petri dishes when their moisture content declined. All the experiments were laid out in a completely randomized design in a factorial arrangement replicated four times and were repeated in time.

### Observations

Germination of seeds was recorded on daily basis according to AOSA^29^. Seed was considered to be germinated when radicle length exceeded 2 mm. Germination percentage was taken as the ratio of number of seeds germinated to the total number of seeds sown and is expressed as percentage. Germination index (GI) was calculated as described by AOSA^30^. 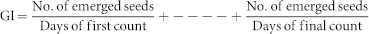


Vigor index was calculated by multiplying germination index with seedling length^31^. Shoot and root length of ten randomly selected seedlings from each replication were measured at 7 DAS. Seedlings of each replicate were dissected into roots and shoots and their fresh weight was recorded immediately.

For determination of α-amylase activity, 1.0 g fresh seedling sample was ground and mixed with 100 ml distilled water, and left for 24 h at 4°C. The enzyme activity was determined from supernatant liquid by dinitrosalicyclic acid (DNS) method^32^. To determine total soluble sugars, ground seedling sample (1 g) was mixed with 10 ml distilled water and left for 24 h at 25°C^33^. Mixture was filtered with Whatman No. 42 (Whatman plc, Kent, UK) and the final volume was made to 10 ml with distilled water. Total soluble sugars were determined by the phenol sulphuric method^34^.

### Statistical analysis

All data from three experiments are presented as the mean value ± standard error (SE) of four replicates. Analyses were performed using the software Statistix 9.0 and the mean variance of the data was analyzed using least significant difference (LSD) test at 0.05 probability level^35^.

## Author Contributions

S.H. and L.N. initiated and designed the research, S.H., Z.M. and F.K. performed the experiments, S.H. and L.N. analyzed the data and wrote the manuscript, A.K., F.S., S.B., J.H. and K.C. revised and edited the manuscript and also provided advice on the experiments.

## Figures and Tables

**Table 1:** Germination percentage, germination index and vigor index of primed rice seeds after 210 days of storage at 25°C or −4°C in Experiment 1

Storage conditions	Priming treatments	Germination percentage	Germination index	Vigor index
**Huanghuazhan**				
0 DAP	NP	87.50 ± 0.83 c†	14.22 ± 0.55 c	116.63 ± 7.05 d
	HP	94.17 ± 0.96 b	22.06 ± 1.69 b	202.99 ± 17.53 c
	PEG	98.33 ± 1.36 a	24.53 ± 0.59 ab	236.13 ± 6.89 ab
	Spd	100.00 ± 0.00 a	25.70 ± 0.95 a	259.04 ± 9.56 a
210 DAP (−4°C)	NP	87.50 ± 0.83 c	14.22 ± 0.55 c	116.63 ± 7.05 d
	HP	93.33 ± 1.36 b	21.76 ± 1.94 b	201.59 ± 19.30 c
	PEG	94.17 ± 0.83 b	22.50 ± 1.45 b	231.65 ± 11.54 b
	Spd	97.50 ± 1.66 a	25.43 ± 0.91 a	251.13 ± 9.81 a
210 DAP (25°C)	NP	80.83 ± 0.83 d	13.26 ± 0.76 c	79.57 ± 4.58 e
	HP	2.50 ± 0.83 e	0.14 ± 0.01 d	0.08 ± 0.01 f
	PEG	5.00 ± 0.96 e	0.42 ± 0.02 d	0.26 ± 0.02 f
	Spd	3.33 ± 1.36 e	0.24 ± 0.01 d	0.12 ± 0.01 f
**Yangliangyou-6**				
0 DAP	NP	89.17 ± 0.83 c	15.65 ± 1.35 d	138.24 ± 14.76 d
	HP	95.83 ± 1.36 ab	21.74 ± 2.57 bc	232.93 ± 26.98 bc
	PEG	96.67 ± 1.36 a	23.33 ± 1.23 ab	250.69 ± 13.66 b
	Spd	100.00 ± 0.00 a	26.27 ±1.42 a	309.86 ± 16.22 a
210 DAP (−4°C)	NP	89.17 ± 0.83 c	15.65 ± 1.35 d	138.24 ± 14.76 d
	HP	94.17 ± 1.66 b	19.69 ± 1.56 c	201.09 ± 19.29 c
	PEG	97.50 ± 0.96 a	23.46 ± 2.60 ab	254.68 ± 12.69 b
	Spd	98.33 ± 1.66 a	24.13 ± 1.18 ab	272.92 ± 32.08 ab
210 DAP (25°C)	NP	80.83 ± 1.59 d	13.28 ± 0.92 d	79.63 ± 6.90 e
	HP	16.67 ± 1.36 f	1.75 ± 0.28 e	3.41 ± 0.33 f
	PEG	25.00 ± 1.66 e	2.97 ± 0.06 e	9.04 ± 0.97 f
	Spd	22.50 ± 0.83 e	2.72 ± 0.11 e	7.15 ± 0.26 f

^†^Different lowercase letters denote statistical differences between treatments of a cultivar at the 5% level according to LSD test. DAP: days after priming, NP: non-primed seeds, HP: hydropriming, PEG: 10% polyethylene glycol priming, Spd: 0.5 mmol L^−1^ spermidine priming, Fresh primed seeds were sown in 0 DAP treatment. Since the germination was constant after 6 days of sowing (DAS), therefore, germination percentage at 6 DAS has been presented.

**Table 2:** Starch metabolism in primed rice seeds (0 DAS) and seedlings (7 DAS) after 210 days of seed storage at 25°C and −4°C in Experiment 1

		α-amylase activity		Total soluble sugars	
Storage conditions	Priming treatments	Dry seeds[mg g^−1^ seed min^−1^]	Rice seedlings[mg g^−1^ FW min^−1^]	Dry seeds[mg g^−1^ seed]	Rice seedlings[mg g^−1^ FW]
**Huanghuazhan**					
0 DAP	NP	7.41 ± 0.19 c†	14.69 ± 0.33 c	7.95 ± 0.34 c	14.69 ± 0.14 c
	HP	8.76 ± 0.16 b	18.89 ± 0.19 ab	12.21 ± 0.23 ab	15.73 ± 0.21 ab
	PEG	9.38 ± 0.18 a	19.15 ± 0.41 ab	12.84 ± 0.22 a	16.39 ± 0.31 a
	Spd	9.44 ± 0.21 a	19.40 ± 0.39 a	13.08 ± 0.19 a	16.47 ± 0.25 a
210 DAP (−4°C)	NP	7.41 ± 0.19 c	14.69 ± 0.33 c	7.95 ± 0.34 c	14.69 ± 0.14 c
	HP	8.53 ± 0.16 b	18.48 ± 0.27 b	11.90 ± 0.20 b	15.17 ±0.20 b
	PEG	9.14 ± 0.18 a	19.09 ± 0.44 ab	12.75 ± 0.24 a	15.90 ± 0.14 ab
	Spd	9.20 ± 0.14 a	19.07 ± 0.26 ab	12.83 ± 0.21 a	16.07 ± 0.23 a
210 DAP (25°C)	NP	7.09 ± 0.33 c	13.83 ± 0.41 c	7.16 ± 0.36 d	13.29 ± 0.33 d
	HP	4.52 ± 0.19 d	4.77 ± 0.18 d	4.89 ± 0.21 e	6.45 ± 0.26 e
	PEG	5.07 ± 0.19 d	5.46 ± 0.24 d	5.51 ± 0.23 e	7.23 ± 0.27 e
	Spd	4.81 ± 0.23 d	5.09 ± 0.21 d	5.21 ± 0.23 e	6.93 ± 0.18 e
**Yangliangyou-6**					
0 DAP	NP	8.07 ± 0.14 c	15.40 ± 0.14 c	8.16 ± 0.14 c	14.33 ± 0.52 cd
	HP	9.10 ± 0.23 b	19.40 ± 0.41 ab	12.70 ± 0.24 b	15.08 ± 0.29 bc
	PEG	9.69 ± 0.18 a	20.05 ± 0.44 a	13.43 ± 0.25 a	16.38 ± 0.46 a
	Spd	9.73 ± 0.18 a	20.20 ± 0.49 a	13.49 ± 0.36 a	16.30 ± 0.33 a
210 DAP (−4°C)	NP	8.07 ± 0.14 c	15.40 ± 0.14 c	8.16 ± 0.14 c	14.33 ± 0.52 cd
	HP	9.07 ± 0.17 b	19.10 ± 0.39 b	12.52 ± 0.13 b	14.70 ± 0.27 c
	PEG	9.50 ± 0.22 ab	19.85 ± 0.45 a	12.86 ± 0.15 b	15.63 ± 0.26 b
	Spd	9.61 ± 0.15 a	20.03 ± 0.22 a	12.96 ± 0.32 ab	15.43 ± 0.17 b
210 DAP (25°C)	NP	7.50 ± 0.15 d	14.43 ± 0.19 d	7.98 ± 0.21 c	13.83 ± 0.23 d
	HP	4.58 ± 0.27 e	4.83 ± 0.27 e	5.02 ± 0.11 d	6.78 ± 0.25 e
	PEG	4.96 ± 0.09 e	5.40 ± 0.21 e	5.47 ± 0.18 d	6.93 ± 0.21 e
	Spd	4.74 ± 0.18 e	5.13 ± 0.22 e	5.20 ± 0.19 d	6.85 ± 0.35 e

^†^Different lowercase letters denote statistical differences between treatments of a cultivar at the 5% level according to LSD test. DAP: days after priming, NP: non-primed seeds, HP: hydropriming, PEG: 10% polyethylene glycol priming, Spd: 0.5 mmol L^−1^ spermidine priming, DAS: days after sowing. Fresh primed seeds were sown in 0 DAP treatment.

**Table 3:** Germination percentage, germination index and vigor index of primed rice seeds after 0, 15, 30, 45, and 60 days of storage at 25°C in Experiment 2

Storage duration	Priming treatments	Germination percentage	Germination index	Vigor index
**Huanghuazhan**				
0 DAP	NP	88.89 ± 1.11 b†	21.68 ± 2.34 bcd	211.63 ± 21.95 cd
	HP	98.89 ± 1.11 a	26.29 ± 2.97 a	268.71 ± 27.42 ab
	PEG	100.00 ± 0.00 a	27.86 ± 1.26 a	285.18 ± 12.58 a
	Spd	100.00 ± 0.00 a	28.06 ± 2.17 a	308.72 ± 22.90 a
15 DAP	HP	96.67 ± 1.93 a	23.63 ± 2.25 abc	212.02 ± 23.75 cd
	PEG	98.89 ± 1.11 a	25.21 ± 1.35 ab	240.35 ± 10.95 bc
	Spd	100.00 ± 0.00 a	24.89 ± 0.62 ab	265.38 ± 5.50 ab
30 DAP	HP	78.89 ± 1.11 c	14.40 ± 0.26 ef	99.78 ± 1.59 f
	PEG	84.44 ± 1.11 b	18.89 ± 1.54 de	161.24 ± 9.94 e
	Spd	88.89 ± 2.94 b	20.31 ± 2.15 cd	176.26 ± 17.53 de
45 DAP	HP	65.56 ± 2.94 d	12.40 ± 0.47 fg	68.22 ± 1.74 fgh
	PEG	66.67 ± 1.93 d	12.17 ± 0.79 fg	77.41 ± 6.73 fg
	Spd	70.00 ± 0.00 d	14.63 ± 1.28 ef	94.02 ± 12.01 f
60 DAP	HP	45.56 ± 2.22 f	7.01 ± 0.68 h	28.35 ± 2.12 h
	PEG	52.22 ± 4.45 e	8.20 ± 0.97 gh	41.57 ± 3.78 gh
	Spd	54.44 ± 4.01 e	8.47 ± 0.39 gh	40.79 ± 1.44 gh
**Yangliangyou-6**				
0 DAP	NP	90.00 ± 1.93 cd	20.63 ± 2.27 bc	205.01 ± 24.80 c
	HP	100.00 ± 0.00 a	29.04 ± 2.59 a	325.03 ± 28.17 ab
	PEG	100.00 ± 0.00 a	27.96 ± 1.71 a	336.23 ± 23.34 a
	Spd	100.00 ± 0.00 a	29.46 ± 0.55 a	343.77 ± 8.79 a
15 DAP	HP	94.44 ± 1.11 bc	20.85 ± 1.77 bc	217.62 ± 15.14 c
	PEG	97.78 ± 2.22 ab	25.28 ± 2.32 ab	278.95 ± 24.57 b
	Spd	100.00 ± 0.00 a	24.92 ± 3.60 ab	307.97 ± 41.17 ab
30 DAP	HP	83.33 ± 1.93 e	18.53 ± 1.04 cd	166.32 ± 7.98 cd
	PEG	87.78 ± 2.22 de	21.29 ± 1.34 bc	206.10 ± 13.79 c
	Spd	88.89 ± 2.22 d	18.96 ± 1.63 cd	182.50 ± 14.36 c
45 DAP	HP	66.67 ± 3.85 g	11.23 ± 0.43 efg	84.28 ± 4.69 ef
	PEG	75.56 ± 1.11 f	13.31 ± 0.52 ef	111.31 ± 5.98 e
	Spd	76.67 ± 3.34 f	14.95 ± 0.36 de	120.19 ± 0.78 de
60 DAP	HP	51.11 ± 2.94 h	7.10 ± 0.10 g	31.36 ± 1.54 f
	PEG	55.56 ± 2.22 h	8.28 ± 0.64 g	45.92 ± 3.16 f
	Spd	62.22 ± 2.94 g	8.78 ± 0.79 fg	42.89 ± 3.64 f

^†^Different lowercase letters denote statistical differences between treatments of a cultivar at the 5% level according to LSD test. DAP: days after priming, NP: non-primed seeds, HP: hydropriming, PEG: 10% polyethylene glycol priming, Spd: 0.5 mmol L^−1^ spermidine priming. Fresh primed seeds were sown in 0 DAP treatment, while in NP, non-primed seeds stored at −4°C were used for comparison. Since the germination was constant after 6 days of sowing (DAS), therefore, germination percentage at 6 DAS has been presented.

**Table 4:** Variations in starch metabolism of primed rice seeds (0 DAS) and seedling (7 DAS) in relation to the duration of storage (25°C) in Experiment 2

		α-amylase activity		Total soluble sugars	
Storage duration	Priming treatments	Dry seeds[mg g^−1^ seed min^−1^]	Rice seedlings[mg g^−1^ FW min^−1^]	Dry seeds[mg g^−1^ seed]	Rice seedlings[mg g^−1^ FW]
**Huanghuazhan**					
0 DAP	NP	7.13 ± 0.29 d†	13.99 ± 0.61 ef	9.43 ± 0.21 de	13.94 ± 0.36 d
	HP	8.30 ± 0.18 bc	19.04 ± 0.65 b	11.80 ± 0.11 bc	16.09 ± 0.11 b
	PEG	9.33 ± 0.16 a	19.87 ± 0.34 ab	12.52 ± 0.32 ab	17.65 ± 0.34 a
	Spd	9.26 ± 0.34 a	20.54 ± 0.37 a	12.76 ± 0.17 a	18.10 ± 0.28 a
15 DAP	HP	8.20 ± 0.11 c	15.93 ± 0.41 d	11.46 ± 0.27 c	15.07 ± 0.29 c
	PEG	8.79 ± 0.34 abc	16.74 ± 0.63 cd	12.08 ± 0.06 abc	16.24 ± 0.11 b
	Spd	8.85 ± 0.30 ab	17.52 ± 0.58 c	12.15 ± 0.29 abc	16.27 ± 0.29 b
30 DAP	HP	7.01 ± 0.03 d	13.12 ± 0.54 f	9.53 ± 0.36 d	11.85 ± 0.33 f
	PEG	7.32 ± 0.41 d	14.22 ± 0.59 e	9.95 ± 0.12 d	12.79 ± 0.21 e
	Spd	7.01 ± 0.27 d	14.34 ± 0.57 e	9.92 ± 0.23 d	12.94 ± 0.25 e
45 DAP	HP	5.86 ± 0.07 ef	9.92 ± 0.39 h	8.05 ± 0.14 fgh	8.89 ± 0.24 gh
	PEG	6.26 ± 0.32 e	11.20 ± 0.31 gh	8.71 ± 0.21 ef	9.27 ± 0.37 gh
	Spd	6.07 ± 0.27 e	12.12 ± 0.39 fg	8.47 ± 0.26 fg	9.58 ± 0.45 g
60 DAP	HP	4.76 ± 0.26 g	6.61 ± 0.21 i	6.94 ± 0.26 i	7.83 ± 0.26 i
	PEG	5.78 ± 0.31 ef	7.69 ± 0.23 i	7.87 ± 0.24 gh	8.62 ± 0.43 hi
	Spd	5.34 ± 0.33 fg	6.70 ± 0.15 i	7.66 ± 0.11 hi	7.97 ± 0.19 i
**Yangliangyou-6**					
0 DAP	NP	7.86 ± 0.31 de	17.38 ± 0.21 de	9.82 ± 0.27 e	14.52 ± 0.14 ef
	HP	9.24 ± 0.25 ab	20.55 ± 0.38 b	12.47 ± 0.24 ab	18.06 ± 0.21 ab
	PEG	9.53 ± 0.34 a	21.44 ± 0.64 a	12.48 ± 0.13 ab	18.18 ± 0.26 a
	Spd	9.85 ± 0.17 a	21.85 ± 0.27 a	12.83 ± 0.19 a	18.30 ± 0.35 a
15 DAP	HP	8.39 ± 0.16 c	18.36 ± 0.33 cd	11.93 ± 0.11 bc	16.52 ± 0.08 d
	PEG	8.54 ± 0.21 bc	18.32 ± 0.56 cd	12.38 ± 0.43 abc	16.99 ± 0.25 cd
	Spd	8.98 ± 0.21 b	19.27 ± 0.17 c	12.69 ± 0.27 a	17.46 ± 0.33 bc
30 DAP	HP	7.61 ± 0.31 e	15.19 ± 0.62 g	11.05 ± 0.59 d	13.84 ± 0.48 f
	PEG	8.05 ± 0.28 cd	15.97 ± 0.54 fg	11.73 ± 0.27 c	14.69 ± 0.36 e
	Spd	8.28 ± 0.23 c	16.70 ± 0.41 ef	11.91 ± 0.07 bc	14.78 ± 0.11 e
45 DAP	HP	6.07 ± 0.29 f	11.97 ± 0.40 h	9.14 ± 0.35 efg	10.31 ± 0.41 h
	PEG	6.57 ± 0.31 f	12.63 ± 0.40 h	9.53 ± 0.19 ef	11.43 ± 0.33 g
	Spd	6.39 ± 0.24 f	12.23 ± 0.38 h	9.42 ± 0.31 ef	11.38 ± 0.37 g
60 DAP	HP	5.23 ± 0.19 g	7.56 ± 0.30 k	7.78 ± 0.09 h	8.21 ± 0.17 j
	PEG	6.21 ± 0.24 f	8.99 ± 0.17 j	8.98 ± 0.06 fg	9.58 ± 0.12 hi
	Spd	5.55 ± 0.17 g	8.03 ± 0.57 jk	8.69 ± 0.29 g	9.02 ± 0.28 ij

^†^Different lowercase letters denote statistical differences between treatments of a cultivar at the 5% level according to LSD test. DAP: days after priming, NP: non-primed seeds, HP: hydropriming, PEG: 10% polyethylene glycol priming, Spd: 0.5 mmol L^−1^ spermidine priming, DAS: days after sowing. Fresh primed seeds were sown in 0 DAP treatment, while in NP, non-primed seeds stored at −4°C were used for comparison.

**Table 5:** Effect of re-priming and heating on germination percentage and starch metabolism in primed rice seeds after 210 days of storage at 25°C in Experiment 3

Post-storage treatments	Pre-storage treatments	Germination percentage	α-amylase activity[mg g^−1^ FW min^−1^]	Total soluble sugar[mg g^−1^ FW]
**Huanghuazhan**				
Control (un-treated)	NP	82.50 ± 2.10 a	13.94 ± 0.13 a	11.83 ± 0.39 a
	HP	1.67 ± 0.96 b	4.83 ± 0.13 b	6.95 ± 0.19 b
	PEG	3.33 ± 1.36 b	5.34 ± 0.07 b	7.69 ± 0.18 b
	Spd	1.67 ± 0.96 b	5.04 ± 0.13 b	7.45 ± 0.22 b
Re-priming	HP	3.33 ± 1.36 b	4.86 ± 0.06 b	7.05 ± 0.11 b
	PEG	4.17 ± 1.60 b	5.27 ± 0.15 b	7.66 ± 0.22 b
	Spd	2.50 ± 0.83 b	5.18 ± 0.09 b	7.53 ± 0.13 b
Heating at 45°C	HP	0.83 ± 0.83 b	5.04 ± 0.22 b	7.06 ± 0.39 b
	PEG	2.50 ± 0.83 b	5.16 ± 0.23 b	7.68 ± 0.45 b
	Spd	2.50 ± 0.83 b	5.29 ± 0.31 b	7.50 ± 0.34 b
**Yangliangyou-6**				
Control (un-treated)	NP	85.00 ± 2.15 a	14.88 ± 0.15 a	12.41 ± 0.34 a
	HP	18.33 ± 0.96 b	6.02 ± 0.23 b	8.46 ± 0.20 b
	PEG	20.83 ± 0.83 b	6.44 ± 0.14 b	9.03 ± 0.42 b
	Spd	20.83 ± 1.60 b	6.31 ± 0.32 b	8.88 ± 0.36 b
Re-priming	HP	19.17 ± 1.60 b	6.07 ± 0.25 b	8.50 ± 0.23 b
	PEG	21.67 ± 1.67 b	6.39 ± 0.16 b	8.98 ± 0.20 b
	Spd	22.50 ± 0.83 b	6.41 ± 0.17 b	9.02 ± 0.30 b
Heating at 45°C	HP	18.33 ± 1.67 b	6.10 ± 0.22 b	8.45 ± 0.14 b
	PEG	23.33 ± 1.36 b	6.41 ± 0.10 b	9.02 ± 0.30 b
	Spd	21.67 ± 0.96 b	6.38 ± 0.11 b	8.96 ± 0.14 b

^†^Different lowercase letters denote statistical differences between treatments of a cultivar at the 5% level according to LSD test. NP: non-primed seeds, HP: hydropriming, PEG: 10% polyethylene glycol priming, Spd: 0.5 mmol L^−1^ spermidine priming. In re-priming treatment, primed rice seeds after 210 days of storage were re-treated with same priming agent. Heat treatment was given for 24 hours. Since the germination was constant after 6 days of sowing (DAS), therefore, germination percentage at 6 DAS has been presented. Total soluble sugars and α-amylase activity were measured after 7 days of sowing. In NP, dry seeds were also stored at 25°C for 210 days.
